# Supplementary figures and images for: Using micro-CT to explore bone density variations in the skulls of the vulnerable Opsariichthys uncirostris uncirostris (Three-lips fish) during reproductive migration to a Lake Biwa tributary
Source: PLoS One. 2024 Nov 11;19(11):e0310461. doi: 10.1371/journal.pone.0310461 (PMC11554045; doi:10.1371/journal.pone.0310461)

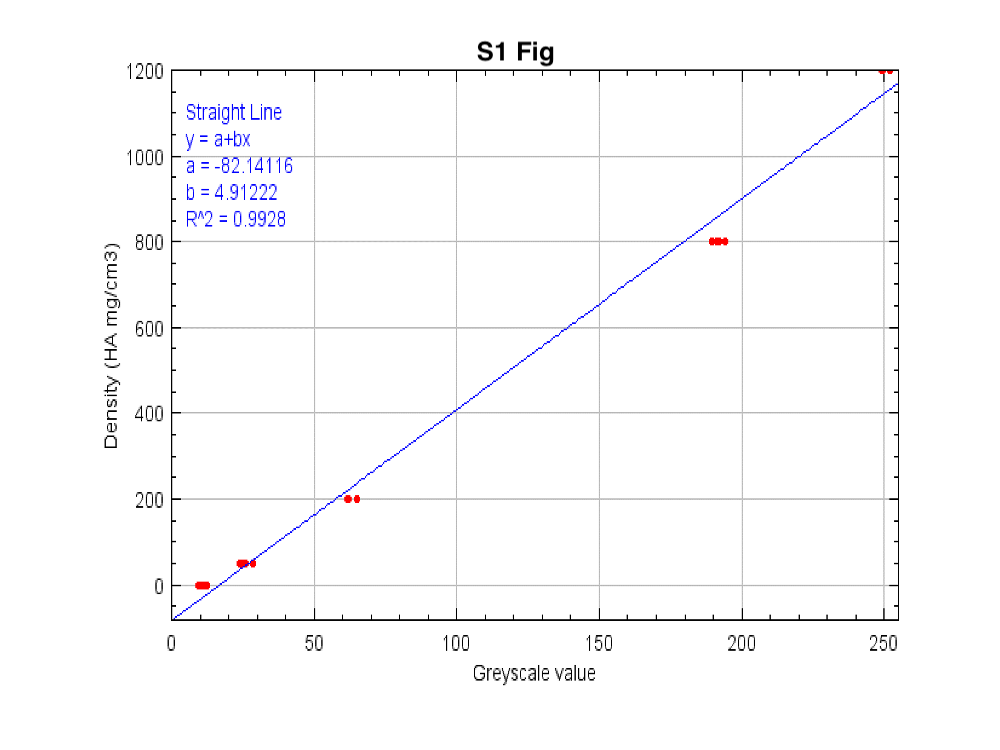

Supplement: S1 Fig — (TIF) [file pone.0310461.s001.tif]
