# Supplementary material for: Using micro-CT to explore bone density variations in the skulls of the vulnerable Opsariichthys uncirostris uncirostris (Three-lips fish) during reproductive migration to a Lake Biwa tributary
Source: PLoS One. 2024 Nov 11;19(11):e0310461. doi: 10.1371/journal.pone.0310461 (PMC11554045; doi:10.1371/journal.pone.0310461)
Supplement: S2 File — (PDF) [file pone.0310461.s003.pdf]

```
#R script for micro-CT  paper on Three-lips fish

####Loading Packages####
library(ggplot2)
library(dplyr)
library(vioplot)
library(ggbeeswarm)
library(ggpubr)
library(scales)
library(gridExtra)
library(tidyverse)
library(NSM3)
library(PMCMR)

####Loading data####
CT<-read.csv("S1_File.csv", header=T)
CT<-as.data.frame(CT)

####Visualizations####
#Length vs density
CTLength<-ggplot(CT, aes(y=Density..HA.mean., x=SL_.mm.,col=Sex,fill=Sex,linetype=Sex)) +
  geom_point(shape=21,size=2, alpha=0.5,col="black")+
  geom_smooth(method = "lm", se = F,lwd=0.8)+
  labs(x='Standard length (mm)',y=expression('Relative bone density [CaHA (mg cm'^-1*')]'))+coord_cartesian(xlim =
c(150,300),ylim=c(500,750))+
  theme_bw()+
  scale_fill_manual(values = alpha(c('blue', 'orange'), 0.5), breaks = c('M', 'F'), name = 'Sex') +
  scale_color_manual(values = c('black', 'black'), breaks = c('M', 'F'), name = 'Sex') +
  scale_linetype_manual(values = c('M' = 'solid', 'F' = 'dotted'), breaks = c('M', 'F')) +
  theme(text = element_text(size = 16,family='Helvetica'),plot.margin=unit(c(0.1,-0.008,-0.1,0.1),"cm"),panel.grid.major =
element_blank(),axis.text=element_text(colour="black"),
        panel.grid.minor = element_blank(),panel.border = element_rect(colour='black'),legend.position = 'bottom')
#tiff('Figure_2.tiff',width=180,height=180,units="mm",res=300,pointsize = 16,family='Helvetica')
CTLength
#annotate_figure(CTLength, top = text_grob("Density (HA) vs standard length distribution", face = "bold",family='Helvetica'))
dev.off()

#Condition Factor (K) vs density
CTK <- ggplot(CT, aes(y = Density..HA.mean., x = Condition_Factor, color = Sex, fill = Sex, linetype = Sex)) +
  geom_point(size = 2, shape = 21, alpha = 0.5, color = 'black') +
  geom_smooth(method = "lm", aes(color = Sex), se = F,lwd=0.8) +
  labs(x = 'Condition factor (K)', y = '') +
  coord_cartesian(xlim = c(1, 2), ylim = c(500, 750)) +
  theme_bw() +
  scale_fill_manual(values = alpha(c('blue', 'orange'), 0.5), breaks = c('M', 'F'), name = 'Sex') +
  scale_color_manual(values = c('black', 'black'), breaks = c('M', 'F'), name = 'Sex') +
  scale_linetype_manual(values = c('M' = 'solid', 'F' = 'dotted'), breaks = c('M', 'F')) +
  theme(text = element_text(size = 16, family = 'Helvetica'),plot.margin = unit(c(0.1, 0.2, -0.1, -1), "cm"),panel.grid.major =
element_blank(),axis.text = element_text(colour = "black"),
    axis.text.y = element_blank(),panel.grid.minor = element_blank(),panel.border = element_rect(colour = 'black'),legend.position =
'bottom')
#tiff('Figure_1.tiff',width=180,height=180,units="mm",res=300,pointsize = 16,family='Helvetica')
CTK
#annotate_figure(CTK, top = text_grob("Density (HA) vs Condition Factor (K)", face = "bold",family='Helvetica'))
dev.off()

tiff('Fig2.tiff',width=180,height=180,units="mm",res=300,pointsize = 16,family='Helvetica')
Com<-ggarrange(CTLength,CTK,common.legend = T,legend='bottom',align = 'hv')
annotate_figure(Com, top = text_grob("Fig 2", face = "bold",family='Helvetica'))
dev.off()
Com

#Bone density changes across reproductive season
Seasonal_changes<-CT %>% filter(Month!="Jun"|Sex=="M",) %>%
  ggplot(aes(x=factor(Month, levels=c("Jun","Jul","Aug","Sep")),y=Density..HA.mean.,fill=factor(Sex, levels=c('M','F'))))+
  scale_fill_viridis_d(option='D')+
  geom_violin(alpha=0.25,position = position_dodge(width=0.75),lwd=0.5,col='black')+
  ggbeeswarm::geom_quasirandom(shape = 21,size=2, dodge.width = .75, color="black",alpha=0.5,show.legend = T)+
  annotate('point',x=1.3,y=CT$Density..HA.mean.[CT$Month=='Jun'& CT$Sex=='F'],shape=21,size=2,col='black',fill='orange',alpha=0.5)+
  labs(x='Sampling month',y=expression('Relative bone density [CaHA (mg cm'^-1*')]'))+coord_cartesian(ylim=c(500,750))+
  scale_fill_manual(name='Sex',values=c('blue','orange'),breaks=c('M','F'))+
  theme_bw()+
  theme(text = element_text(size = 16,family='Helvetica'),plot.margin=unit(c(0.1,0.1,0.1,0.1),"cm"),panel.grid.major =
element_blank(),axis.text=element_text(colour="black"),
        panel.grid.minor = element_blank(),panel.border = element_rect(colour='black'),legend.position = 'bottom')
tiff('Fig3.tiff',width=180,height=180,units="mm",res=300,pointsize = 16,family='Helvetica')
Seasonal_changes
annotate_figure(Seasonal_changes, top = text_grob("Fig 3", face = "bold",family='Helvetica'))
dev.off()

####Statistical analysis####
#GLM with guassian family (LM)
CTlm<-
glm(Density..HA.mean.~SL_.mm.+Condition_Factor+Sex+SL_.mm.*Condition_Factor*Sex+SL_.mm.*Condition_Factor+Sex*SL_.mm.+Condition_Factor*Sex,data
= CT,family =
            "gaussian")
plot(CTlm)
summary(stepAIC(CTlm)) #Table 1
plot(stepAIC(CTlm))


#Kruskal-wallis & pairwise tests
##For all individuals##
#Selecting Jul-Aug due to low  sample size  in Jun & Aug for Females
Denstest<-subset(CT, !(Month %in% c("Jun","Sep")))
#KW test
kruskal.test(Density..HA.mean.~interaction(Sex,factor(Month, levels=c("Jul","Aug"))),data=Denstest)
#Pairwise test
pSDCFlig(Denstest$Density..HA.mean.,interaction(Denstest$Sex,factor(Denstest$Month, levels=c("Jul","Aug"))),method="Monte Carlo")

##For males only##
#Selecting Males only
```

```
DenstestM<-subset(CT, !(Sex %in% c("F")))
#Selecting Jun-Aug due to sample size in Sep
DenstestM2<-subset(DenstestM, !(Month %in% c("Sep")))

#KW test
kruskal.test(Density..HA.mean.~factor(Month, levels=c("Jun","Jul","Aug")),data=DenstestM2)
#Pairwise test
pSDCFlig(DenstestM2$Density..HA.mean.,factor(DenstestM2$Month, levels=c("Jun","Jul","Aug")),method="Monte Carlo")
```
