# Supplementary material for: Using micro-CT to explore bone density variations in the skulls of the vulnerable Opsariichthys uncirostris uncirostris (Three-lips fish) during reproductive migration to a Lake Biwa tributary
Source: PLoS One. 2024 Nov 11;19(11):e0310461. doi: 10.1371/journal.pone.0310461 (PMC11554045; doi:10.1371/journal.pone.0310461)
Supplement: Attachment — Submitted filename: Response_to_editor.docx [file pone.0310461.s004.docx]

Dear Editor

We wish to submit a revised version of our original research article entitled “Using micro-CT to explore bone density variations in the skulls of the vulnerable *Opsariichthys uncirostris uncirostris* (Three-lips fish) during reproductive migration to a Lake Biwa tributary” for consideration by the Plos One journal.

Re: Resubmission of revised manuscript No. PONE-D-24-06945

Thank you for inviting us to submit a revised draft of our original manuscript entitled, “Using micro-CT to explore bone density variations in the skulls of the vulnerable *Opsariichthys uncirostris uncirostris* (Three-lips fish) during reproductive migration to a Lake Biwa tributary” to the Plos One journal.

We would like to express our sincere gratitude to you and the editor for the time and effort you have dedicated to reviewing the manuscript. The valuable feedback has greatly contributed to improving the quality of the manuscript.

Revisions in the manuscript are shown as track changes. Overall, we have specified the permits obtained for fish sampling in our study. We have also updated the “S1 File” to reference [18] after the manuscript underling the data was accepted to Ichthyological Research. Below this letter we address each of the concerns raised by the editor.

Thank you once again for considering our manuscript for publication in the Plos One journal. We look forward to your feedback on the revised version and hope for a favorable outcome.

Sincerely,

Andrew Mvula

PhD Student, Department of Environmental Solution Technology

Ryukoku University

Response to editor

Concern 1.01: Permits and approvals obtained for the work.

Response 1.01: The following statement has been added in L.120 p6 of the manuscript: “Fish sampling in the Shiotsuo River was done with approval from the Department of Fisheries of Shiga Prefecture (permit number: 30-34, flag numbers: 274, 454 and 537).”

Concern 1.02: Whether the land accessed is privately owned or protected.

Response 1.02: The following statement has been added in L.104 p5 of the manuscript: “The study was conducted in the lower reaches of the Shiotsuo River (under the control of the Shiga Prefectural local government)…”

Concern 1.03: Whether any protected species were sampled.

Response 1.03: No protected species were sampled in this study.

Concern 1.04: Full details of animal husbandry, experimentation, and care/welfare, where relevant.

Response 1.04: Not applicable in this study.
