# Supplementary material for: Using micro-CT to explore bone density variations in the skulls of the vulnerable Opsariichthys uncirostris uncirostris (Three-lips fish) during reproductive migration to a Lake Biwa tributary
Source: PLoS One. 2024 Nov 11;19(11):e0310461. doi: 10.1371/journal.pone.0310461 (PMC11554045; doi:10.1371/journal.pone.0310461)
Supplement: Attachment — Submitted filename: Response to Reviewers.docx [file pone.0310461.s005.docx]

Dear Prof. Arnar Palsson,

We wish to submit a revised version of our original research article entitled “Using micro-CT to explore bone density variations in the skulls of the vulnerable *Opsariichthys uncirostris uncirostris* (Three-lips fish) during reproductive migration to a Lake Biwa tributary” for consideration by the Plos One journal.

Re: Resubmission of revised manuscript No. PONE-D-24-06945

Thank you for inviting us to submit a revised draft of our original manuscript entitled, “Using micro-CT to explore bone density variations in the skulls of the vulnerable *Opsariichthys uncirostris uncirostris* (Three-lips fish) during reproductive migration to a Lake Biwa tributary” to the PLOS ONE journal.

We would like to express our sincere gratitude to you, the reviewers and the editor for the time and effort dedicated to reviewing the manuscript. The valuable feedback has greatly contributed to improving the quality of the manuscript.

Revisions in the manuscript are shown as track changes (line numbers mentioned are from the revised manuscript with all mark-up and revisions inline). Overall, we have formatted the manuscript according to the PLOS ONE templates. The data underlying this study has also been included as supplementary material to the submission (including R script). We would also like to clarify that "The funders had no role in study design, data collection and analysis, decision to publish, or preparation of the manuscript." The reference list has also been reviewed and two new references [8, 23, 25] have been added to the manuscript after revesion. Finally, the figures have also been checked using the PACE digital diagnostic tool. Below this letter we address each of the concerns raised by you, the reviewers and other editors.

Thank you once again for considering our manuscript for publication in the Plos One journal. We look forward to your feedback on the revised version and hope for a favorable outcome.

Sincerely,

Andrew Mvula

**Responses to specific concerns**

**1.00 Journal Requirements**

Concern 1.01: Please ensure that your manuscript meets PLOS ONE's style requirements, including those for file naming.

Response 1.01: The manuscript has been modified in accordance to PLOS ONE’s style requirements provided in the templates.

Concern 1.02: Please note that PLOS ONE has specific guidelines on code sharing for submissions in which author-generated code underpins the findings in the manuscript. In these cases, all author-generated code must be made available without restrictions upon publication of the work.

Response 1.02: The R code underlying the findings in the manuscript has been shared as supporting information (S2_File.R).

Concern 1.03: Please state what role the funders took in the study. If the funders had no role, please state: "The funders had no role in study design, data collection and analysis, decision to publish, or preparation of the manuscript."

Response 1.03: The role of the funders has been mentioned in the rebuttal letter as follows: “The funders had no role in study design, data collection and analysis, decision to publish, or preparation of the manuscript.”

Concern 1.04: In the online submission form, you indicated that "The data underlying the findings of this study may be made available upon reasonable request from the corresponding author."

Response 1.04: The data underlying this study (S1_File.csv) has been provided as supplemtary material in the submission.

Concern 1.05: Please review your reference list to ensure that it is complete and correct. If you have cited papers that have been retracted, please include the rationale for doing so in the manuscript text, or remove these references and replace them with relevant current references. Any changes to the reference list should be mentioned in the rebuttal letter that accompanies your revised manuscript. If you need to cite a retracted article, indicate the article’s retracted status in the References list and also include a citation and full reference for the retraction notice.

Response 1.05: The references have been checked and ensured it is complete and correct. Where necessary, any changes have been mentioned in the rebuttal letter.

**2.00 Major points**

Concern 2.01: Note PLOS-ONE has strict data sharping policy. Data have to be made accessible. Use data.dryad.org, Figshare or comparable.

Response 2.01: The data underlying the findings in this study (S1_File.csv) has been attached as supporting material to the submission.

Concern 2.02: The experimental design is a bit unclear, explain better when the fish are migrating, and if only migrating fish were caught (no controls?). This could be iterated in the results section, update statements about monhts with points about when migrating and when not.

Response 2.02: The period when the fish are migrating has been clarified in the materials and methods section as follows: from L144 (revised manuscript with track changes) “…a preferred reproductive upstream migration route for Three-lips fish when they seasonally migrate from May to September. Reproductive migration of Three-lips fish in Lake Biwa has been documented in several studies using conventional and environmental DNA (eDNA) analysis [22-23]. A study on δ^13^C and δ^15^N stable isotopes in muscle and mucus tissues of Three-lips fish during their reproductive migration also indicates that the fish may migrate and inhabit spawning sites at varying times, with some individuals staying longer than others in the Shiotsuo River [19].”. In addition, a statement on controls was added as follows: from L154 (revised manuscript with track changes) “Since the fish were caught during their reproductive migration, no control specimens were obtained.”

Concern 2.03: The influence of sex is not very convincing. This was not supported by the linear models. Check the results and dicussion from line 228 onwards. You can retain the discussion on potential differences, but don’t overstate the results.

Response 2.03: The section on influence of sex has been modified to not overstate results in the “Sex roles potentially influence bone density in Three-lips fish” section as follows: from L307 (revised manuscript with track changes) “Results from the bone density analysis also indicated that there are potential differences between male Three-lips fish and female Three-lips fish with males having a relatively lower bone density than females (Figs 2-3)... However, a larger sample size is required to confidently conclude on the effects of sex on bone density in Three-lips fish.”

Concern 2.04: The discussion should be split into three sections, add a section on the month differences (migrating period), and move the section on “putative sex differences” down. See also abstract and conclusions.

Response 2.04: The discussion has been split into three sections as suggested: i) effect of age and environment on bone density, ii) bone density distribution across the Three-lips migrating period and iii) sex roles potentially influence bone density in Three-lips fish.

**3.00 Intermediate points**

Concern 3.01: Line 51. “The assessment of the fish skeletal system, such as bone density, not only offers a unique perspective in determining the intricacies of fish physiology, and but also provides insights into the health status of individuals”I would say that the skeletal system primarlity informs about function, feeding and mobility adaptations, physiology and health are then secondary.

Response 3.01: The statement has been modified as follows: from L59 (revised manuscript with track changes) “Although the skeletal system primarily informs function, feeding and mobility adaptations, the assessment of the fish skeletal system, such as bone density, can also offer a unique perspective in determining the intricacies of fish physiology, and provide insights into the health status of individuals.”

Concern 3.02: Line 65 “subsequent reproduction [2, 7].” Are there other ways to evaluate this relationship? Mention complementary methods, here or in discussion.

Response 3.02: The complementary methods have been mentioned in the introduction section as follows: from L75 (revised manuscript with track changes) “A number of tools can be used to evaluate this relationship including, biochemical markers (e.g., C-terminal telopeptide), nutritional analyses, hormonal analyses and computed tomography. Selecting the right tools for evaluation not only enhances our comprehension of the life history strategies of potamodromous fish but also provides a valuable tool for assessing the impact of anthropogenic activities on critical migratory corridors, ultimately contributing to the conservation and sustainable management of these essential fish populations…”

Concern 3.03: Line 96-Clarify the aims of study, and provide one or more specific research questions or hypotheses.

Response 3.03: The specific research questions have been stated as follows: from L112 (revised manuscript with track changes) “The specific research question for this study were as follows: is the bone density of the cranium in Three-lips fish related to size and health of the fish? Are there differences in the bone density of the carnium in Three-lips individuals sampled in different months during their reproductive season? Are there bone density differences in the carnium of male and female Three-lips individuals sampled during their reproductive migration?”

Concern 3.04: Lines 106-115. Description of system is lengthy. What in this is really needed for this study, the RQ, results and discussion? These fish migrate, is there seasonality in that or not??

Response 3.04: We feel it is important to describe the syetem in detail because it paints a picture of the reproductive migration journey the Three-lips fish undertakes. On the other concerns, the element of seasonality has been clarified as follows: from L124 (revised manuscript with track changes) “…a preferred reproductive upstream migration route for Three-lips fish when they seasonally migrate from May to September. Reproductive migration of Three-lips fish in Lake Biwa has been documented in several studies using conventional and environmental DNA (eDNA) analysis [22-23]. A study on δ^13^C and δ^15^N stable isotopes in muscle and mucus tissues of Three-lips fish during their reproductive migration also indicates that the fish may migrate and inhabit spawning sites at varying times, with some individuals staying longer than others in the Shiotsuo River [19].” The expected results have also been stated as follows: from L139 (revised manuscript with track changes) “It is expected that Three-lips fish caught at different times during their reproductive migration in Shiotsuo River will have varying bone density in their cranium due to size and health, time the species was caught as well as sex of the individual.”

Concern 3.05: Lines 185- Clarify description, the “negative relationship” applies to the quantatitaive variables, but does not hold meaning for the sex differences. Split into two sentences, say explicitly Sex did not have a sign. effect. Also, not necessary to use the word “biometrics”, rather use explanatory variable or similar. Also the expectation in line 187, are bigger fish thought to be more healthy?

Response 3.05: The statements have been modified as follows to address the concerns: from L219 (revised manuscript with track changes) “The GLM model with gaussian family and stepAIC revealed that the relative bone density decreased as the explanatory variables standard length, condition factor increased and a tendency for lower bone density in males when compared to females (Table 1). The standard length and condition factor (*K*) had significant effects on the relative bone density in the GLM (*p* < 0.05) while sex did not have a significant effect on the relative bone density (*p* > 0.05). The result of the GLM on condition factor (*K*) was contrary to the expectation that healthier individuals, regardless of size, would have a larger bone density than slightly less healthier individuals.”

Concern 3.06: Line 205. Your overall model did not support this, but still you conclude… “These results indicate that females have higher densities when compared to males during the reproductive season.” I don’t think the data support this conclusion. Rather that the sex effects were small if real, bigger sample needed to confirm. Use in discussion also.

Response 3.06: The statement has been modified as follows: from L247 (revised manuscript with track changes) “These results indicate that there may be differences in bone density due to sex, however, a bigger sample size is need to confirm this observation.”

Concern 3.07: Line 210- Frame the discussion better. Mention the organism, and iterate why comparison of months is important. It is a bit unclear if all the fish are migrating, or if only fish in some months are migrating??

Response 3.07: The discussion section has been modified to reflect the concerns. In particular the discussion section has been split into three sections as follows: i) effect f age and environment on bone density, ii) bone density distribution across the Three-lips migrating period and iii) sex roles potentially influence bone density in Three-lips fish. The organism has also been mentioned. In addition, a previous study, that clarifies if or some fish are migrating (using stable isotopes) has been cited as follows: from L127 (revised manuscript with track changes) “A study on δ^13^C and δ^15^N stable isotopes in muscle and mucus tissues of Three-lips fish during their reproductive migration also indicates that the fish may migrate and inhabit spawning sites at varying times, with some individuals staying longer than others in the Shiotsuo River [19].”

Concern 3.08: Line 265. Other future studies could also be envisioned. Can you propose a replicate of this study, that can more thoroughly answer the research questions? More sampling, telemetry, isotopes ect???

Response 3.08: The following studies has been envisioned: from L295 (revised manuscript with track changes) “However, there is a need for further investigations on the behaviors of Three-lips fish before and after the peak migration period to draw conclusions on which scenario is more likely. For example, future studies may consider exploring the immediate impact of various stimuli (e.g., mechanical and chemical stimuli) on bone density in Three-lips fish. Furthermore, the bone density analysis in this study was done on the cranium as a whole, however, future studies should assess bone density differences in individual bones of the cranium. For example, these studies could focus on the differences in bone density between the dentaries and premaxilla. This would not only provide insights into the dynamic nature of Three-lips fish bone remodeling in response to varying environments but could also inspire the development of new biotechnological approaches for bone tissue engineering and regeneration in humans, as is the case with zebrafish and other marine species [1, 41].”

Concern 3.09: Conclusions, Line 272 -“In conclusion, this study demonstrated variations in bone densities between male and female” not supported strongly by data. Start with the result most strongly supported. Also the statement “the distinct changes in bone density during each catch are likely because of resource mobilization strategies employed by Three-lips fish to ensure successful reproduction. Can this be concluded from the data? Build this argument better in discussion! Concluding sentence could be more direct, “about the influence of migration, changes in physiology etc on bone density variation”…

Response 3.09: The conclusion has been modified as follows: from L355 (revised manuscript with track changes) “In conclusion, this study demonstrated variations in bone densities in Three-lips fish of different ages and health during their reproductive migration to a Lake Biwa tributary. Although not significantly different, the study also highlighted possible differences between male and female Three-lips fish, with females potentially having higher bone density than males. We recognize the limitations in this study and that the results must be treated with caution due to the relatively low sample size. Nevertheless, the findings from this study serve as a foundation for ecologists and biomechanics hoping to study the effects of migration, changes in physiology and age on bone density changes in Three-lips fish and other species in various ecosystems.”

**4.00 Minor points**

Concern 4.01: Line 56. Drop mention of “this study”, make the statement more general.

Response 4.01: The statement has been modified as follows: from L65 (revised manuscript with track changes) “Integrating bone density analysis in the study of the potamodromous fish has potential to unveil a unique dimension linked to the reproductive migration of the species.”

Concern 4.02: Line 72. Add “of”. “Understanding of bone density variations in fish”

Response 4.02: from L81 (revised manuscript with track changes) “of” has been added to read “understanding of…”

Concern 4.03: Line 76 and 77. “drastic” used twice. Replace or drop.

Response 4.03: the sentence has been modified as follows: from L89 (revised manuscript with track changes) “…identify drastic changes in fish energetics especially during the reproductive season when weight changes can be observed due to reproductive behavior”

Concern 4.04: Line 79. micro-CT is not novel, maybe “newly developed”? When was this developed??

Response 4.04: from L91 (revised manuscript with track changes) “novel” has been changed to “newly developed”.

Concern 4.05: Line 83. “Images” not “imaging”

Response 4.05: The statement already has images and not imaging.

Concern 4.06: Line 89. Drop “allows us to”

Response 4.06: from L101 (revised manuscript with track changes) “allows us to” has been dropped from the statement.

Concern 4.07: Line 104. Study was not completed in the river, the samples came from the river. Rewrite.

Response 4.07: from L120 (revised manuscript with track changes) The statement has been modified as follows: “The samples in this study were collected in the lower reaches of the Shiotsuo River…”

Concern 4.08: Line 123. Each month? Better than sampling, also give total sampled in study “(total XX fish)”.

Response 4.08: The statement has been modified as follows to reflect the concerns: from L148 (revised manuscript with track changes) “The sampling was systematic and a target of 20 Three-lips individuals was set for each month (i.e., 20 individuals x 5 months to collect a total of 100 individuals). Despite sampling efforts, however, no individuals were caught in the month of May, only 12 individuals were caught in the month of June and only 5 individuals were caught in the month of September (i.e., only 57 individuals were collected from the planned 100 for the entire sampling period).”

Concern 4.09: Line 133. Replace “After the 48 hrs. had elapsed,” with “Next”

Response 4.09: from L166 (revised manuscript with track changes) “After the 48 hrs. had elapsed” has been replaced with “Next”.

Concern 4.10: Line 179. Simplify to. “sufficient sample size for”

Response 4.10: from L213 (revised manuscript with track changes) The statement has been modified as follows: “…due to a sufficient sample size for statistical analysis.

Concern 4.11: Line 191. Drop “(estimates ± standard errors)” from table title. Also, the estimate between sexes, is that male minus female or other way around? Explain in either title or footnote.

Response 4.11: from L229 (revised manuscript with track changes) “(estimates ± standard errors)” has been dropped from the table title. The following footnote has also been added to the table from L233 (revised manuscript with track changes) “^a^The estimate for "Sex" in the linear regression model reflects the effect on the dependent variable specifically for males compared to females, using a dummy variable approach.”

Concern 4.12: Line 200. Perhaps “pairwise comparison revealed” instead of “the comparisson test revelead”??

Response 4.12: from L240 (revised manuscript with track changes) “the comparisson test revealed” has been replaced by “pairwise comparison revealed”.

Concern 4.13: Line 215. Drop “are”?? “reproductive season are fall within the”

Response 4.13: from L257 (revised manuscript with track changes) “are” has been dropped from the statement.

Concern 4.14: Line 219. Could rather than would

Response 4.14: from L262 (revised manuscript with track changes) “would” has been replaced by “could”

Concern 4.15: Line 222. Drop “to” in front of “the negative relationship between”

Response 4.15: from L264 (revised manuscript with track changes) “to” has been dropped in front of “the negative relationship between”

Concern 4.16: Line 224. Drop “muscle” - “gonads” is the main deal?

Response 4.16: from L266 (revised manuscript with track changes) “muscle” has been dropped. The statement reads as follows: “…perhaps to other tissue such as gonads which play a more direct role in spawning”

Concern 4.17: Line 246. Reword “migration” …”fewer fish are migrating could” – and drop “as”

Response 4.17: from L273 (revised manuscript with track changes) The statement has been reworded as follows: “…when fewer individuals are migrating could be a result of one of three scenarios…”

Concern 4.18: Line 247. Drop “likely”

Response 4.18: from L274 (revised manuscript with track changes) “likely” has been dropped from the statement.

Concern 4.19: Line 249. Citing the figure does not make sense here, it only shows data, and does not explain these models.

Response 4.19: from L275 (revised manuscript with track changes) the figure citation has been removed.

Concern 4.20: Line 249. “due to the reduced numbers,” what does this refer to? Fewer fish?

Response 4.20: from L278 (revised manuscript with track changes) The statement has been modified as follows: “…possible that due to fewer fish in the river,…”

Concern 4.21: Lome 252. Reword “new individuals approaching the”, “new individuals enter the”

Response 4.22: from L278 (revised manuscript with track changes) The statement has been modified as follows: “…possible that the higher bone density in June and September is because of new individuals entering…”

Concern 4.22: Line 255. Reword, not sure what this means “journeys and this is not reflected in the changes in bone”

Response 4.22: from L283 (revised manuscript with track changes) The statement has been reworded as follows: “It is likely that these individuals, during the early and late migration waves, have enough energy reserves in tissues other than bones to undertake upstream reproductive migration. As such, no drastic changes in bone density are expected.”

Concern 4.23: Line 432. Fig2 legend. Simplify to “Relationship between relative bone density and (a) standard length and (b) condition factor.” And “Solid lines indicate the linear fit for the relationships”.

Response 4.23: from L539 (revised manuscript with track changes) Fig 2. legend has been reworded as follows: “Fig 2. Relationship between relative bone density and (a) standard length and (b) condition factor. The blue and orange points indicate male and female Three-lips fish, respectively. The solid lines indicate the linear fit for the relationships.”

**5.00 Reviewers' comments**

Concern 5.01:Methods. how were fish sacrificed in the field? Did this comply with permitting standards?

Response 5.01: The following statement has been added on sacrificing fish: from L152 (revised manuscript with track changes) “The fish were sacrificed by placing them in ice water and in compliance with Japanese laws and standards.”

Concern 5.02: Methods. How did you determine sex in the three lips fish?

Response 5.02: The following statement has been added on sex determination: from L156 (revised manuscript with track changes) “The sex of the fish was confirmed in the field by dissecting the fish and checking the gonads for sperm and eggs.”

.

Concnern 5.03: Methods. please identify which bones in the skull were used in the density measurements. Are there density differences at the level of individual bones or is the density similar across all facial bones?

Response 5.03: In this study all bones in the cranium were used in the density measurements. This has been modified in the materials and methods under measuring relative bone density in skulls of Three-lips fish as follows from L180 (revised manuscript with track changes) “The cranium (skull bones) in the head scans were segmented…”. We do acknoweldge that there could be possible differences between bones in the skull. However, this study did not assess individual bone differences. Instead, we offer a recommendation to assess individual bones in the discussion section as follows from L298 (revised manuscript with track changes) “Furthermore, the bone density analysis in this study was done on the cranium as a whole, however, future studies should assess bone density differences in individual bones of the cranium. For example, these studies could focus on the differences in bone density between the dentaries and premaxilla.”

Concern 5.04: are there bone size differences in addition to density differences?

Response 5.04: We reckon there are bone size differences in addition to the density differences as evident from the SL variation in the total catch.

Concnern 5.05: Line 53: “skeletogenesis of fish is a progressive” should read “skeletogenesis of fish is progressive…”

Response 5.05: from L62 (revised manuscript with track changes) “a” has been removed from the statement.

Concern 5.06: Line 143: cite 3D Slicer: Fedorov A., Beichel R., Kalpathy-Cramer J., Finet J., Fillion-Robin J-C., Pujol S., Bauer C., Jennings D., Fennessy F.M., Sonka M., Buatti J., Aylward S.R., Miller J.V., Pieper S., Kikinis R. 3D Slicer as an Image Computing Platform for the Quantitative Imaging Network. Magnetic Resonance Imaging. 2012 Nov;30(9):1323-41. PMID: 22770690. PMCID: PMC3466397.

Response 5.06: 3D slicer has been cited from L455 (revised manuscript with track changes): 25. Fedorov A, Beichel R, Kalpathy-Cramer J, Finet J, Fillion-Robin J-C, Pujol S, et al. 3D Slicer as an Image Computing Platform for the Quantitative Imaging Network. Magnetic Resonance Imaging. 2012 Nov;30(9):1323-41. PMID: 22770690. PMCID: PMC3466397.

Concern 5.07: Line 214: remove “are” from the sentence.

Response 5.07: from L257 (revised manuscript with track changes) “are” has been removed from the sentence.
