# Supplementary material for: Using micro-CT to explore bone density variations in the skulls of the vulnerable Opsariichthys uncirostris uncirostris (Three-lips fish) during reproductive migration to a Lake Biwa tributary
Source: PLoS One. 2024 Nov 11;19(11):e0310461. doi: 10.1371/journal.pone.0310461 (PMC11554045; doi:10.1371/journal.pone.0310461)
Supplement: Attachment — Submitted filename: Response to Reviewers.docx [file pone.0310461.s006.docx]

Dear Prof. Arnar Palsson,

We wish to submit a revised version of our original research article entitled “Using micro-CT to explore bone density variations in the skulls of the vulnerable *Opsariichthys uncirostris uncirostris* (Three-lips fish) during reproductive migration to a Lake Biwa tributary” for consideration by the Plos One journal.

Re: Resubmission of revised manuscript No. PONE-D-24-06945R1

Thank you for inviting us to submit a revised draft of our original manuscript entitled, “Using micro-CT to explore bone density variations in the skulls of the vulnerable *Opsariichthys uncirostris uncirostris* (Three-lips fish) during reproductive migration to a Lake Biwa tributary” to the PLOS ONE journal.

We would like to express our sincere gratitude to you, the reviewers and the editor for the time and effort dedicated to reviewing the manuscript. The valuable feedback continues to improves the clarity and quality of the manuscript.

Revisions in the manuscript are shown as track changes (line numbers and pages mentioned are from the revised manuscript with all mark-up and revisions inline). Overall, we have also explained why the correlated variables (standard length and condition factor) were not assessed monthly in this study. We have also used consistent colors to indicate sexes in the different figures. The reference list has also been reviewed and a new reference for the R programming software has been added. Finally, grammatical errors have also been addressed. Below this letter we address each of the concerns raised by you, the reviewers and other editors.

Thank you once again for considering our manuscript for publication in the Plos One journal. We look forward to your feedback on the revised version and hope for a favorable outcome.

Sincerely,

Andrew Mvula

**Responses to specific concerns**

1.0 **Journal requirements**

Concern 1.01: Please review your reference list to ensure that it is complete and correct.

Response 1.01: The reference list has been reviewed and ensured it is complete and correct.

**2.00 Key concerns**

Concern 2.01: A key question is whether the correlated variables (length and conditition factor) also differ by months? Please provide a similar analyses as in Figure 3 for the correlates. Are these month differences in bone density are driven by size or condition variation?

Response 2.01: Regarding the differences between length and condition factor across different months has already been assessed using the same biometric data as well as stable isotopes by Mvula & Maruyama 2024. A brief explanation on these changes has been added in L.215 p.11 with reference to the paper as follows “It is worth mentioning that the changes in standard length and condition factor (K) of Three-lips fish across different months have already been documented by Mvula and Maruyama [19]. In their paper, both the standard length and condition factor decreased during peak reproductive migration (July to August), which was attributed to environmental changes, rising water temperatures, and potential alterations in food availability. To effectively and statistically assess the effects of monthly variations in standard length and condition factor (K) on bone density, a larger sample size than the one currently obtained for this study is required.”. As to whether the differences in bone density are driven by size or condition variation, this has already been shown in Table 2 using linear modelling but to effectively assess the monthly differences affect bone density then a larger sample size than currently obtained for this study is required.

Concern 2.02: Also, use consistent colours to indicate the sexes in different figures. Consolidate and standardize legends and caption texts about the graphs also.

Response 2.02: Colors in the figures have been revised and are consitent for the sexes. The caption and legend texts have also been standardized.

**3.00 Minor points**

Concern 3.01: Line 63. Drop “progressive“ Such a progressive process

Response 3.01: L.51 P.4, Progressive has been dropped from the statement.

Concern 3.02: Line 70. Tone down bombastic wording “The interplay between bone density and reproductive migration holds profound ecological significance.”

Response 3.02: L.58 P.4, The statement has been revised as follows: “The relationship between bone density and reproductive migration has ecological significance.”

Concern 3.03: Line 88. Split “cannot”

Response 3.03: L.75 P.5, the word “cannot” has been split to “can not”.

Concern 3.04: Line 90. Drop “the” in front of bone densitiy

Response 3.04: L.77 P.5, the has been dropped in front of bone density.

Concern 3.05: Line 112. Make plural (multiple questions), drop “specific”. “The specific research question for this”

Response 3.05: L.99 P.6, the statement has been revised as follows “The research questions for this study were as follows…”

Concern 3.06: Line 116.Reword „“carnium of male and female Three-lips individuals sampled during” to “cranium of males and females during”

Response 3.06: L.102 P.6, The statement has been reworded as follows “Are there bone density differences in the cranium of males and females during their reproductive migration?”

Concern 3.07: Line 130.“with some individuals staying longer than others in the Shiotsuo River“ Clarify, is this the river they spawn in?

Response 3.07: L.116 p.7, the statement has been revised as follows “A study on δ^13^C and δ^15^N stable isotopes in muscle and mucus tissues of Three-lips fish during their reproductive migration to the Shiotsuo River also indicates that the fish may migrate and inhabit spawning sites at varying times, with some individuals staying longer than others in the river”

Concern 3.08: Line135.This reference to Aye fish is not needed - drop “...as well as Ayu fish. Ayu fish serves as the primary food source for Three-lips fish in the Lake Biwa ecosystem [198, 2432].”

Response 3.08: L.122 p.7, the reference to Ayu fish has been dropped from the statement.

Concern 3.09: Line 148.Refer to this as a “plan”. Reword “20 individuals x 5 months to collect a total of” to “20 individuals over 5 months, in total 100“

Response 3.09: L.134 p.7, the statement has been revised as follows “The sampling plan was systematic and a target of 20 Three-lips individuals was set for each month (i.e., 20 individuals over 5 months, in total of 100).”

Concern 3.10: Line 150-153. Shorten “Despite… ” And refer to a table about sample sizes, sex ratio etc. Use info from line 208- can simplify that section also.

Response 3.10: L.136 p.8, the statement has been revised as follows “Despite sampling efforts, only 57 individuals were collected from the planned 100 (Table 1).” A table has also been added.

Concern 3.11: Line 180.Drop “ the head“

Response 3.11: L.169 p.9, “the head” has been dropped from the statement.

Concern 3.12: Line 193. Add reference to R

Response 3.12: L.182 p.10, The reference has been added to R.

Concern 3.13: Line 211. Replace “However…” with, “In parallel, the analyses was done one male..”

Response 3.13: L.200 p.11, the statement has been modified as follows “In parallel, the analysis was done on male individuals only comparing…”

Concern 3.14: Line 225. Reword „“The result of the GLM on condition factor (K)is” to “The negative relationship of condition factor and bone density was contrary“

Response 3.14: L.213 p.11, the statement has bee reworded as suggested.

Concern 3.15: Line 234. Drop “using a dummy variable approach”

Response 3.15: L.229 p.12, the statement has been dropped from the statement.

Concern 3.16: Line 271. This opening sentence has circular logic. Fix.

Response 3.16: L.266 p.14, the statement has been modified as follows “Assessing the Three-lips fish bone density distribution across different months during their reproductive migration helps identify potential variations in bone density that may be influenced by seasonal changes or migration patterns.”

Concern 3.17: Line 277-78. Reword. The word “potential” is used to often

Response 3.17: L.272 p.14, the statement has been reworded as follows “…demonstrated tentative differences in the timing of upstream migration from Lake Biwa to Shiotsuo River with potential early and late migrations within the population.”

Concern 3.18: Line 308. Reword “ from the bone density analysis also indicated that there are potential differences “ to “The results suggested differences “

Response 3.18: L.303 p.15, the statement has been reworeded as follows, “The results suggested differences between male and female Three-lips fish with males having a relatively lower bone density than females”

Concern 3.19: Line 311.Drop “ than females “

Response 3.19: L.2306 p.16, “than females” has been dropped from the statement.

Concern 3.20: Line 358. Drop “ with females potentially having higher bone density than males”

Response 3.20: L.325 p.16, the statement has been dropped.
